# Nursing Hours and Nursing Institutions

**Published:** 1921-01-01

**Authors:** 


### NURSING HOURS AND NURSING INSTITUTIONS.

It would be extremely useful if the Editor of The Hospital, or the Managing Directors of the College, or somebody else who will and can demonstrate the truth, would set out in plain terms how far the principal existing nurses' institutions actually represent and voice the opinions and ambitions of British trained nurses. I am not now referring to any one particular cause, but, if it helps, I will name one—the problem raised by the Minister of Labour's Hours of Employment Bill. The main function of the College of Nursing I conceive to be the voicing of the opinions and ambitions of its members, which are, I understand, twenty thousand in number.

It is a huge army, one which, efficiently generalled, might accomplish great things.

I do not know whether it would be quite accurate to describe the General Nursing Council as a nurses' institution or not. As it exists it is not an elected body, nor is its legal function to voice the nurses, but rather to see that the Registration Acts are rightly carried into operation. Now the extraordinary occurrence that took place on the 10th ultimo at the meeting of the General Nursing Council, and which appears to have passed unnoticed, puzzles me not a little, and causes me to express a hope that some of the authorities will make the truth manifest. After full consideration the College of Nursing submitted a scheme in the form of a schedule for the consideration of the Council. Mrs. Bedford Fenwick moved its rejection, and it was then and there turned down, without discussion.

I consider this a very extraordinary incident, and it can mean only one of two things—either that the College schedule does not represent the views of its members, or else that the Nursing Council is acting in direct opposition to the wishes of the nurses of England and Wales. I am not now discussing the terms of the schedule. They may be of great importance in themselves, but they are a mere detail in the wide question of nurse articulation and representation. If the College, in preparing its schedule, had first made certain that their members approved, it is their immediate duty to point out that the General Council pays no heed to nursing public opinion. If, on the other hand, the College Council have submitted a scheme which they hatched without consulting their members, and which those members would refuse to endorse, they are guilty of a breach of faith of a most serious character. When a Government does this kind of thing, no matter how high the motive, every member thereof has to surrender his portfolio. The College of Nursing is not an autocratic body. By its constitution it is representative, and the members of the Council owe it to their constituents either to interpret their wishes or to abdicate.

### The Schedule.

I am disposed to the opinion that the College authorities framed a schedule which its own members would condemn. I am unable to believe, however hard I try, that any one nurse (never mind twenty thousand) would voluntarily come forward and ask that a fifty-six hours' working week should be substituted for one of forty-eight hours, as suggested by the State. It appears to me an autocratic and reactionary suggestion, and, personally, I am glad that the ladies and gentlemen of the General Nursing Council lit their cigarettes with the schedule. I told Lord Knutsford that a good deal of College literature would go this way if certain changes were not made.

### Keep Down the Nurse!

A fifty-six hours' week means, in a word, no Saturday afternoon and no Sunday. If a nurse is called upon in an emergency to work thus, or even much more, in the vast majority of instances there will be a ready response. Nurses have proved their devotion even unto death. But to propose that nurses shall be segregated from the world's workers in order to impose upon them hours of toil from which all else are by law emancipated—well, it is not exactly what one expects from the nurses' elected representatives! Just think about it for a brief moment. Scavengers, milliners, shop assistants, vanmen, printers, cooks—all and sundry are to have their Saturday afternoons and their Sundays (or their equivalents), but the nurse is to be denied these, and at her own request! The schedule is the product of the nurses' own parliament.

### What Nurses Think.

I am afraid the College Executive fails to realise the trend of public opinion. I have before me a letter signed by "A College Member," who, in the course of a long and somewhat amusing letter, exclaims: "At present if a nurse fails in her fight, all I can say, so far as our own organisations are concerned, heaven help her! They are either too prudent, or blind to the necessity, or yet lack the moral courage, or it might be the fear of getting unpopular." The writer suggests that "a gift of toy artillery" be presented to the Council in order to inspire the martial spirit. The fifty-six-hour schedule appears to me like turning the guns on the nurses themselves. It is Lord Knutsford's famous Charter of Liberty resurrected, and I thought I had helped to officiate at its obsequies.

In the meantime, I venture to suggest that the College Executive and the General Nursing Council should both take themselves to their tents and leave the Minister of Labour in possession. He is the first person in authority to attempt to make nursing a possible profession for British women.

IERNE.

